# Supplementary material for: Microscopy-based phenotypic profiling of infection by Staphylococcus aureus clinical isolates reveals intracellular lifestyle as a prevalent feature
Source: Nat Commun. 2022 Nov 22;13:7174. doi: 10.1038/s41467-022-34790-9 (PMC9684519; doi:10.1038/s41467-022-34790-9)
Supplement: Supplementary file 1 — Supplementary Information [file 41467_2022_34790_MOESM1_ESM.pdf]

# SUPPLEMENTARY FIGURES 1-10

**Microscopy-based phenotypic profiling of infection by *Staphylococcus aureus* clinical isolates reveals intracellular lifestyle as a prevalent feature**

Ines Rodrigues Lopes, Laura Maria Alcantara, Ricardo Jorge Silva, Jerome Josse, Elena Pedrero Vega, Ana Marina Cabrerizo, Melanie Bonhomme, Daniel Lopez, Frederic Laurent, Francois Vandenesch, Miguel Mano, Ana Eulalio

Corresponding authors:

Miguel Mano; mano@ci.uc.pt ; ORCID: 0000-0003-1922-4824

Ana Eulalio; aeulalio@ci.uc.pt ; ORCID: 0000-0002-7355-0674

## Rodrigues Lopes et al. - Supplementary Figure 1

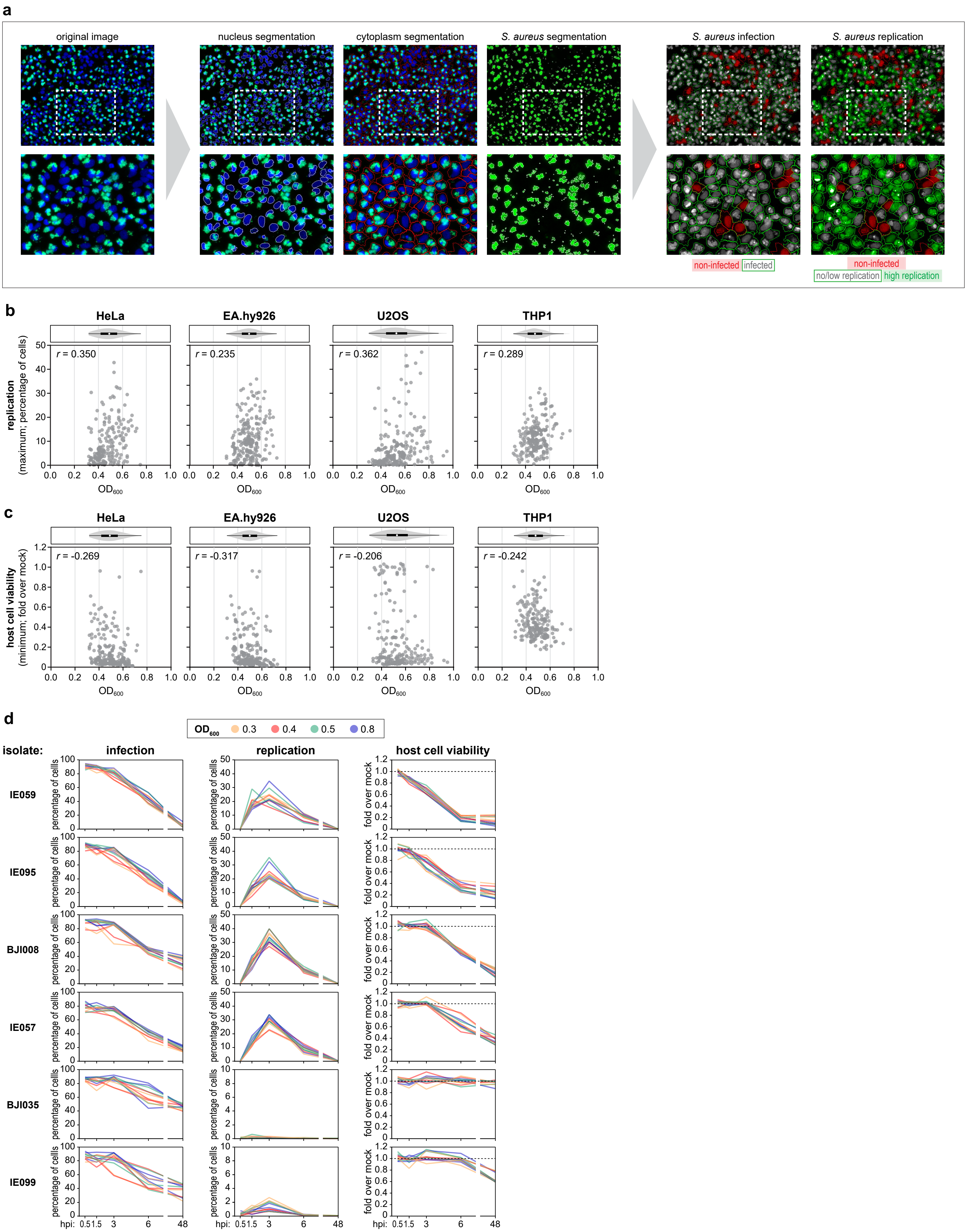

**Supplementary Fig. 1. Analysis of *S. aureus* intracellular lifestyle by fluorescence microscopy-based infection assays followed by automated image analysis.**

**a.** Fluorescence microscopy image, corresponding image segmentation outlines, and classification (infection, replication) of HeLa cells infected with *S. aureus*. In the infection classification panel, cells shaded in red and outlined in green correspond to non-infected and infected cells, respectively; in the replication classification panel, cells shaded in red correspond to non-infected cells, cells outlined in green correspond to cells with no/low *S. aureus* replication and cells shaded in green correspond to cells showing high *S. aureus* replication.

**b and c.** Comparison of $OD_{600}$ of the bacterial growths used for infection and the percentage of host cells with high *S. aureus* intracellular replication (maximum; **b**) or host cell viability (minimum; **c**), for each of the 4 host cell types used (HeLa, EA.hy926, U2OS, and THP1 cells). Results shown for each isolate correspond to the mean of the 3 biologically independent experiments performed. The uppermost plots (violin plots) show the overall distribution of $OD_{600}$ values; white circles show the medians, box limits indicate the $25^{th}$-$75^{th}$ percentiles, whiskers extend 1.5 times the interquartile range from the $25^{th}$ and $75^{th}$ percentiles, polygons extend to extreme values. Spearman's rank correlation coefficients are shown in the upper left corner of each graph.

**d.** Time course analysis of infection, intracellular replication, and host cell viability for selected *S. aureus* isolates in epithelial cells (HeLa). Prior to infection, *S. aureus* isolates were grown to the indicated $OD_{600}$ (0.3, 0.4, 0.5, and 0.8). For each OD and *S. aureus* isolate, the 3 biologically independent experiments are shown.

Source data are provided as a Source Data file.

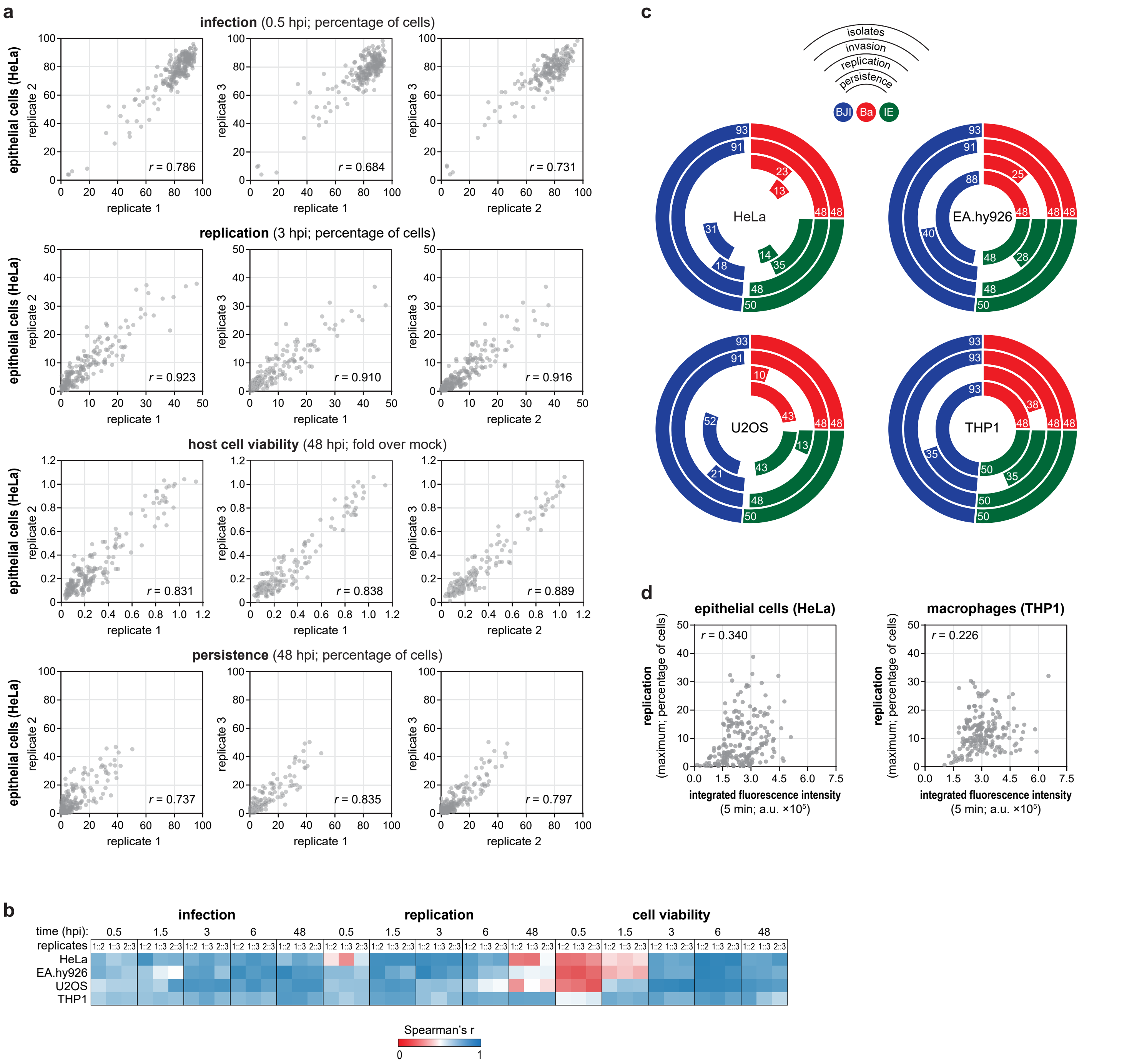


**Supplementary Fig. 2. Readouts from *S. aureus* infection screening show very high reproducibility between the independent experiments.**

**a.** Pairwise correlations between the three independent runs of the screening performed to characterize the infection profile of the 191 *S. aureus* isolates in epithelial cells (HeLa). Results are shown for infection (0.5 hpi), bacterial intracellular replication (3 hpi), host cell viability (48 hpi), and persistence at 48 hpi. Spearman's rank correlation coefficients are shown in the lower right corner of each graph.

**b.** Heat map showing Spearman's rank correlation coefficients between the independent runs of the screening for all phenotypes, cell types, and time-points. Values for Spearman's rank correlation coefficients are included in **Supplementary Data 1**.

**c.** Concentric donut charts showing the number of *S. aureus* isolates showing invasion (infection at 0.5 hpi), high intracellular replication (maximum value), and persistence (infection at 48 hpi) in more than 10% of host cells, for the 4 host cell types tested. Rings are colored according to the clinical origin of the isolates; the total number of isolates per clinical origin is shown in the outermost ring.

**d.** Comparison of the integrated intensity of bacterial fluorescence signal at 5 min pi (proxy for bacterial intracellular load) and the maximum of intracellular replication per isolate, for infection of epithelial cells or macrophages. Spearman's rank correlation coefficients are shown in the upper left corner of each graph.

Source data are provided as a Source Data file.

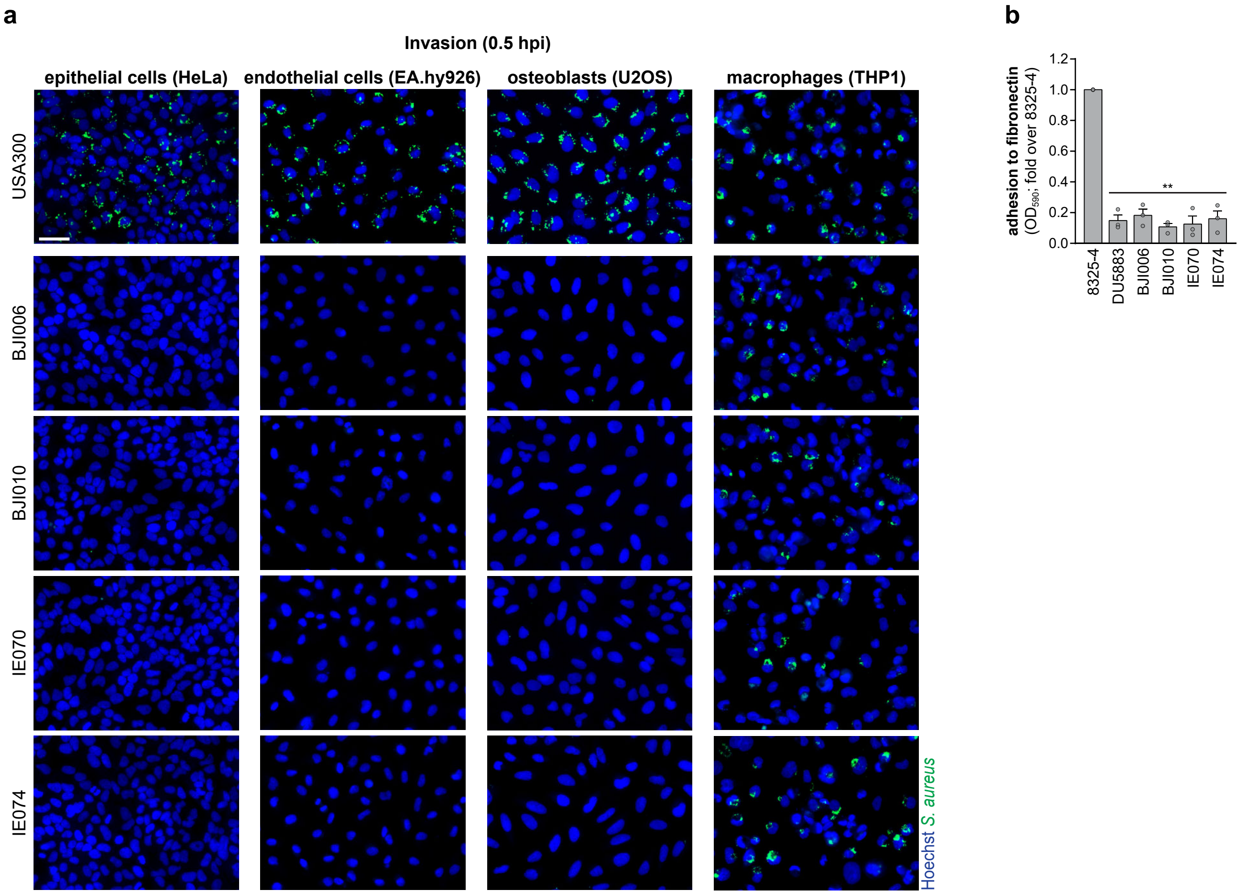


**Supplementary Fig. 3. Four *S. aureus* isolates exhibit very low invasion in non-professional phagocytes due to impaired interaction with fibronectin.**

**a.** Representative fluorescence microscopy images of the four cell types infected with the 4 *S. aureus* isolates identified as exhibiting very low invasion in non-professional phagocytes (HeLa, EA.hy926, and U2OS cells), analyzed at 0.5 hpi. Microscopy images are representative of 3 biologically independent experiments. Scale bar, 50 µm.

**b.** Adhesion of selected *S. aureus* isolates to human fibronectin. Results are shown as mean±s.e.m of 3 biologically independent experiments, normalized to the reference strain *S. aureus* 8325-4; double *fnbA fnbB S. aureus* mutant strain (DU5883) was used for comparison. **P < 0.01 (statistical analysis is detailed in **Supplementary Data 3**).

Source data are provided as a Source Data file.

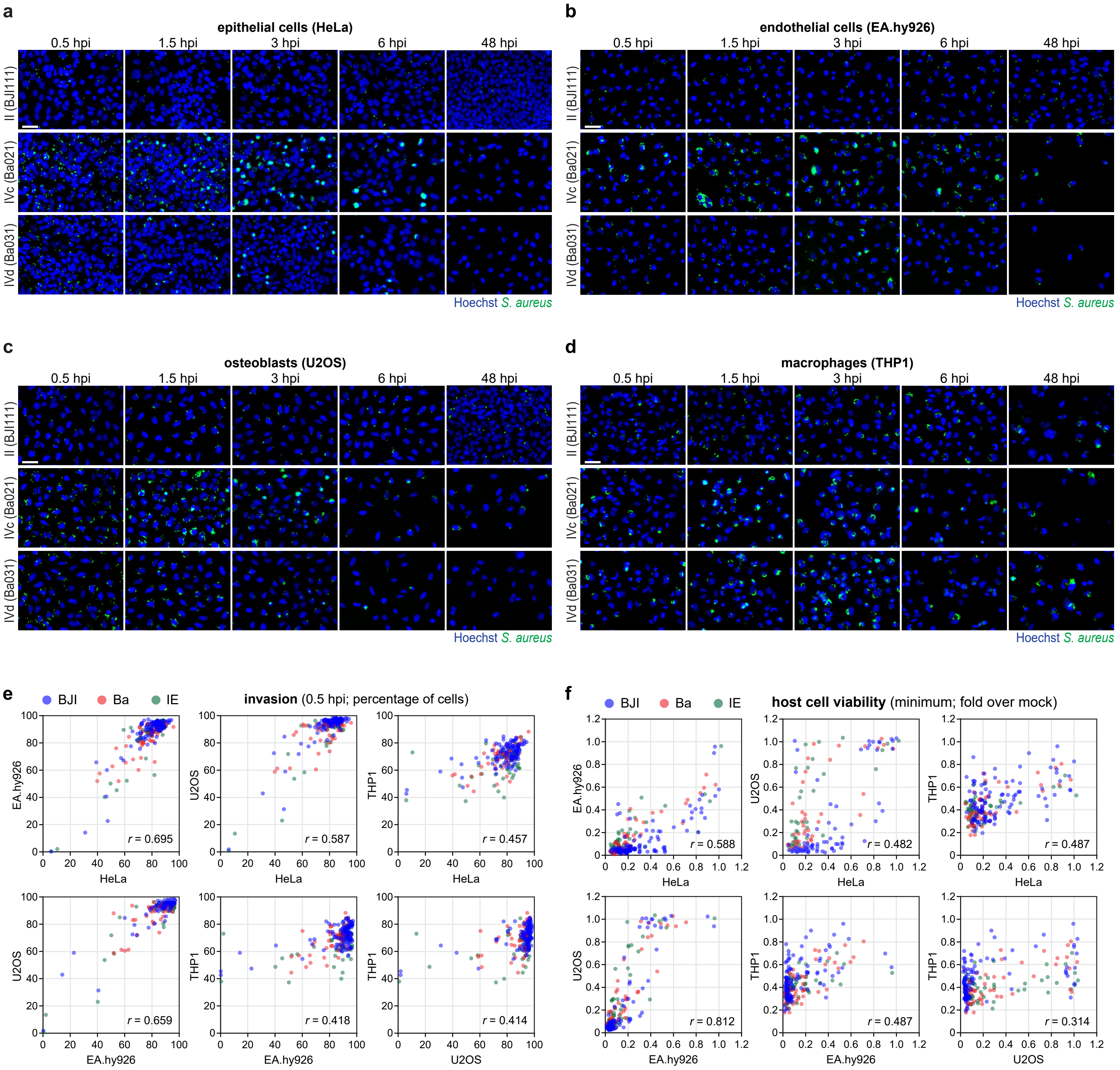


**Supplementary Fig. 4. Intracellular lifestyles of *S. aureus* isolates belonging to distinct phenotypic clusters.**

**a-d.** Representative fluorescence microscopy images of infection of epithelial cells (HeLa; **a**), endothelial cells (EA.hy926; **b**), osteoblasts (U2OS; **c**), and macrophages (THP1; **d**) with selected *S. aureus* isolates belonging to three phenotypic profile clusters identified in **Fig. 2a** (clusters II, IVc, and IVd), at 5 times post-infection (0.5, 1.5, 3, 6, and 48 hpi). Images are representative of 3 biologically independent experiments. Scale bar, 50 µm.

**e and f.** Pairwise comparison of the *S. aureus* invasion (infection at 0.5 hpi; **e**) and host cell viability (minimum value; **f**) upon infection of epithelial cells (HeLa), endothelial cells (EA.hy926), osteoblasts (U2OS), and macrophages (THP1). Colors of datapoints correspond to the clinical origin of the *S. aureus* isolates. Results are presented as the mean of 3 biologically independent experiments. Spearman's rank correlation coefficients are shown in the lower right corner of the graphs.

Rodrigues Lopes et al. - Supplementary Figure 5

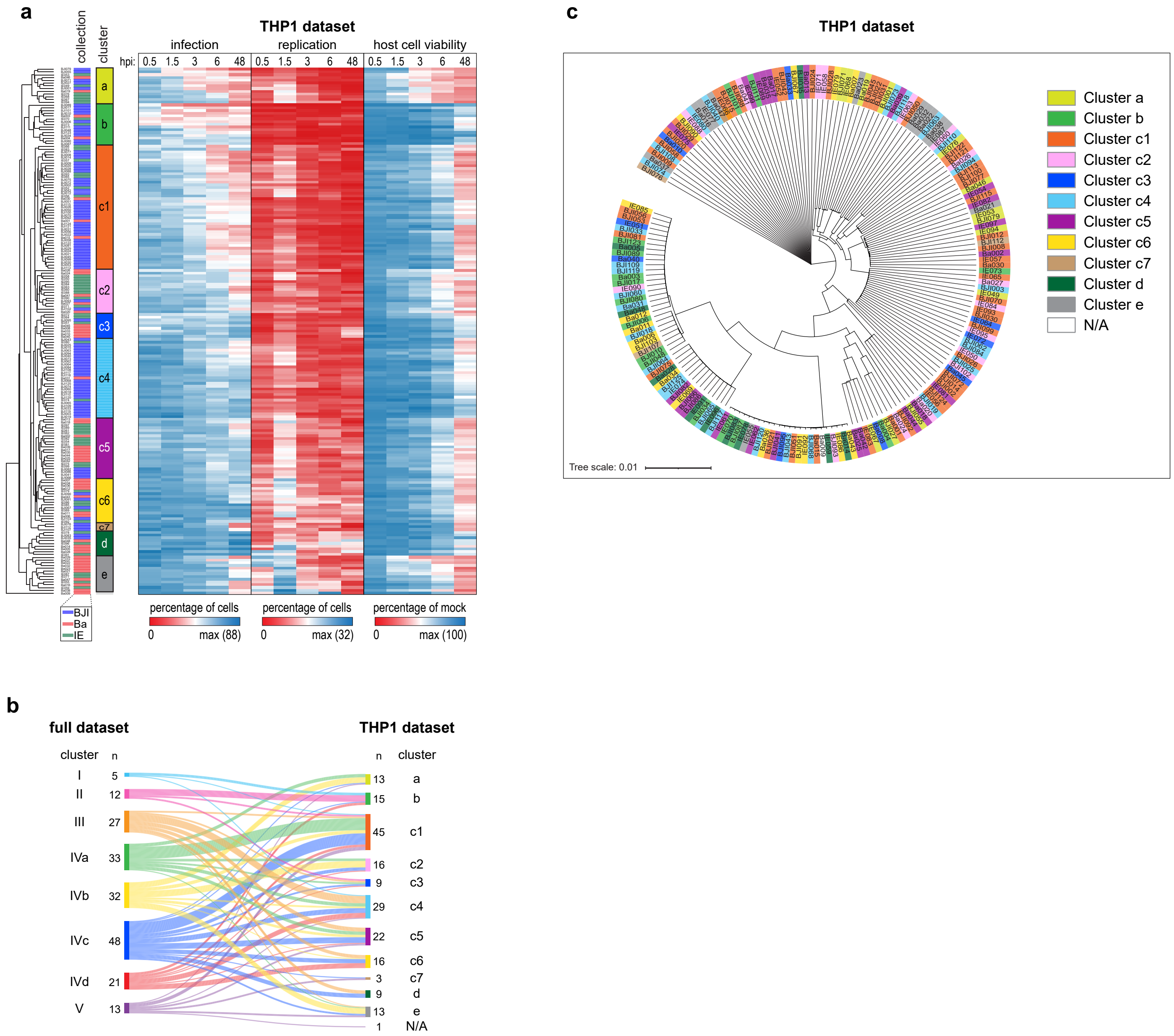


**Supplementary Fig. 5. Clustering based on infection phenotypes in macrophages results in phenotypic groups distinct from those obtained using the full dataset.**

**a.** Heat map showing the phenotypic profiles (infection, intracellular replication, and host cell viability) of the 191 *S. aureus* isolates following infection of phagocytic cells (differentiated THP1) cells, at 5 times post-infection (0.5, 1.5, 3, 6, and 48 hpi). Results are presented as the mean of 3 biologically independent experiments. Hierarchical clustering of the phenotypic profiles exhibited by the bacterial isolates was performed based on Euclidean distance.

**b.** Sankey plot comparing cluster composition based on the phenotypes obtained using the dataset from the 4 cell types (**Fig. 2a**) and that of macrophages (**Supplementary Fig. 5a**).

**c.** Phylogenetic tree of the 191 *S. aureus* clinical isolates based on whole-genome sequencing (core genome). Colors indicate the phenotypic cluster to which each *S. aureus* isolate belongs, based on the hierarchical cluster analysis shown in **Supplementary Fig. 5a**.

**Rodrigues Lopes et al. - Supplementary Figure 6**

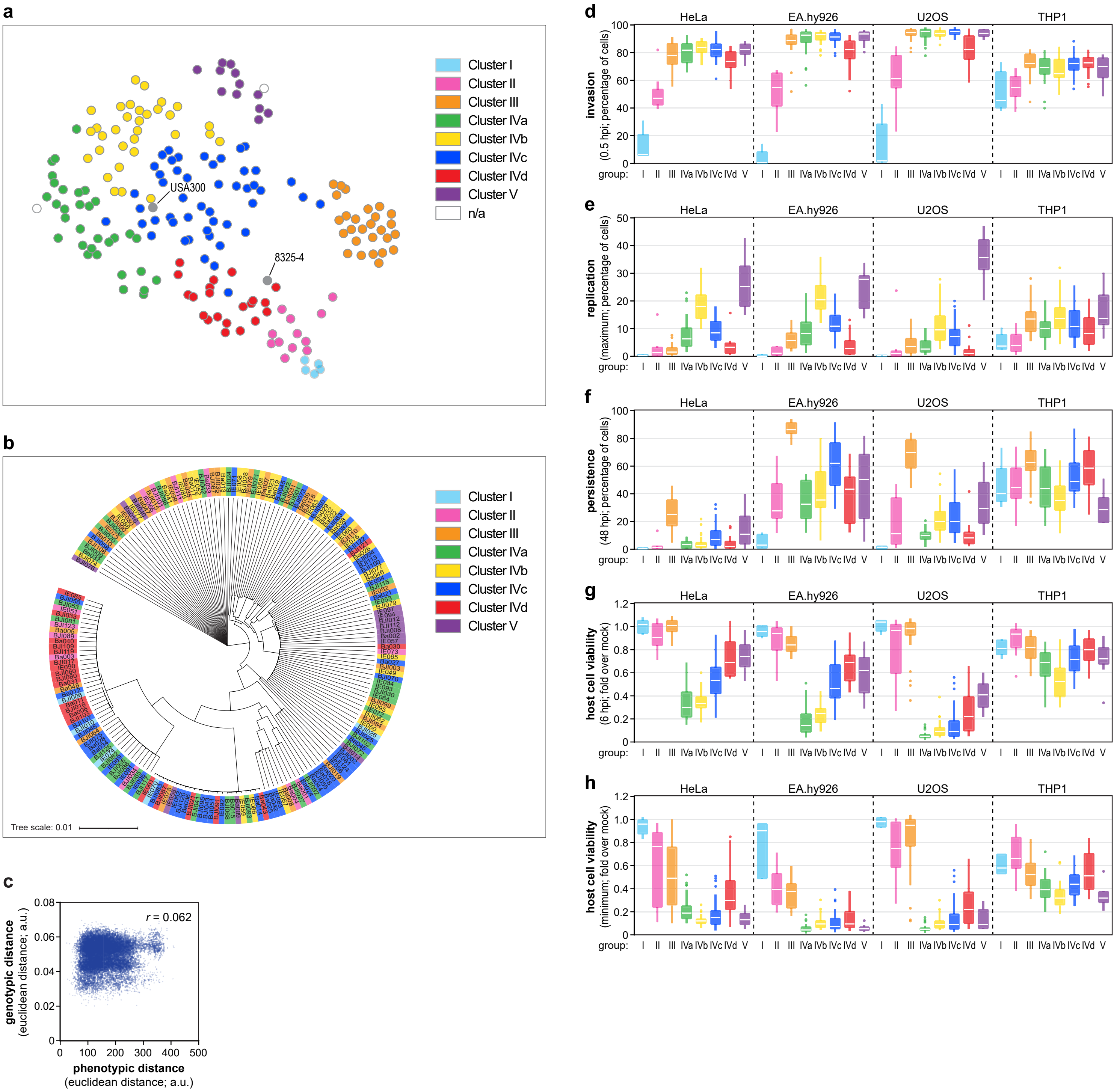


**Supplementary Fig. 6. Infection by *S. aureus* isolates results in distinct intracellular fates.**
**a.** t-SNE visualization of the 191 *S. aureus* isolates based on their individual phenotypic profiles, as described in the legend to **Fig. 4a**. Each circle represents a single *S. aureus* isolate; the circles are colored according to the cluster to which each isolate belongs, as defined by the hierarchical cluster analysis shown in **Fig. 2a**.
**b.** Phylogenetic tree of the 191 *S. aureus* clinical isolates based on whole-genome sequencing (core genome). Colors indicate the phenotypic cluster to which each *S. aureus* isolate belongs, based on the hierarchical cluster analysis shown in **Fig. 2a**.
**c.** Pairwise comparison of the *S. aureus* isolates phenotypic and genotypic distances (Euclidean). Spearman's rank correlation coefficient is shown in the upper right corner.
**d-h.** Box-plots showing the distribution of data for *S. aureus* isolates concerning invasion (infection at 0.5 hpi; **d**), replication (maximum value; **e**), persistence (infection at 48 hpi; **f**), and host cell viability (6 hpi and minimum value; **g and h**). Results are shown for the 4 cell types tested and colored by phenotypic group; box-plots were generated using the mean of 3 biologically independent experiments per clinical isolate; white lines show the medians, box limits indicate the $25^{th}$-$75^{th}$ percentiles, whiskers extend 1.5 times the interquartile range from the $25^{th}$ and $75^{th}$ percentiles. Statistical analysis is detailed in **Supplementary Data 3**.
Source data are provided as a Source Data file.

Rodrigues Lopes et al. - Supplementary Figure 7

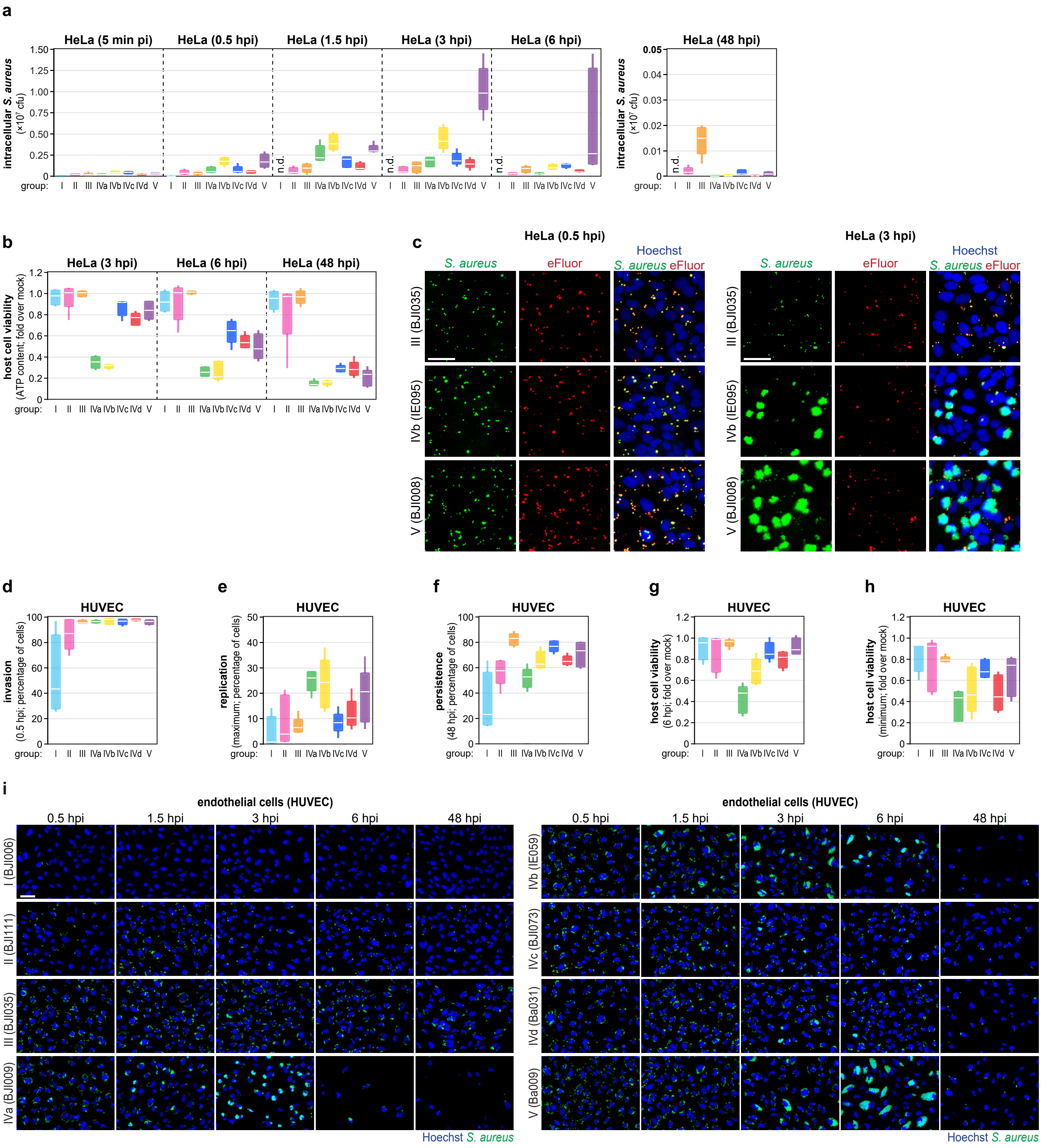

**Supplementary Fig. 7. Infection phenotypes associated with the distinct groups were validated using alternative assays and upon infection of primary cells.**

**a and b.** Box-plots showing the distribution of data for quantification of intracellular *S. aureus* by CFU assays (**a**), and host cell viability determined by luminescence ATP quantification assay (**b**), upon infection of HeLa cells with a subset of bacterial isolates belonging to each phenotypic cluster (4 isolates from cluster I, and 5 isolates from each of the other clusters; total 39 isolates). Infection was analysed at the indicated times; box-plots were generated using the mean of 3 biologically independent experiments per clinical isolate. Results are colored by phenotypic group; white lines show the medians, box limits indicate the $25^{th}$-$75^{th}$ percentiles, whiskers extend 1.5 times the interquartile range from the $25^{th}$ and $75^{th}$ percentiles. Statistical analysis is detailed in **Supplementary Data 3**.

**c.** Representative images of eFluor proliferation assay, upon infection of HeLa cells with 3 *S. aureus* isolates. Infection was analysed at 0.5 and 3 hpi. Microscopy images are representative of 3 biologically independent experiments. Scale bar, 50 µm.

**d-h.** Box-plots showing the distribution of data for infection of primary endothelial cells (HUVEC) with selected *S. aureus* isolates, concerning invasion (infection at 0.5 hpi; **d**), intracellular replication (maximum value; **e**), persistence (infection at 48 hpi; **f**), and host cell viability (6 hpi, and minimum; **g and h**). Infection was performed with a subset of bacterial isolates belonging to each phenotypic cluster (4 isolates from cluster I, and 5 isolates from each of the other clusters; total 39 isolates). Box-plots were generated using the mean of 3 biologically independent experiments per clinical isolate, and are stratified by phenotypic group; white lines show the medians, box limits indicate the $25^{th}$-$75^{th}$ percentiles, whiskers extend 1.5 times the interquartile range from the $25^{th}$ and $75^{th}$ percentiles. Statistical analysis is detailed in **Supplementary Data 3**.

**i.** Representative fluorescence microscopy images of infection of HUVEC with selected *S. aureus* isolates belonging to each phenotypic profile cluster identified in **Fig. 2a**, at 5 times post-infection (0.5, 1.5, 3, 6, and 48 hpi). Images are representative of 3 biologically independent experiments. Scale bar, 50 µm.

Source data are provided as a Source Data file.

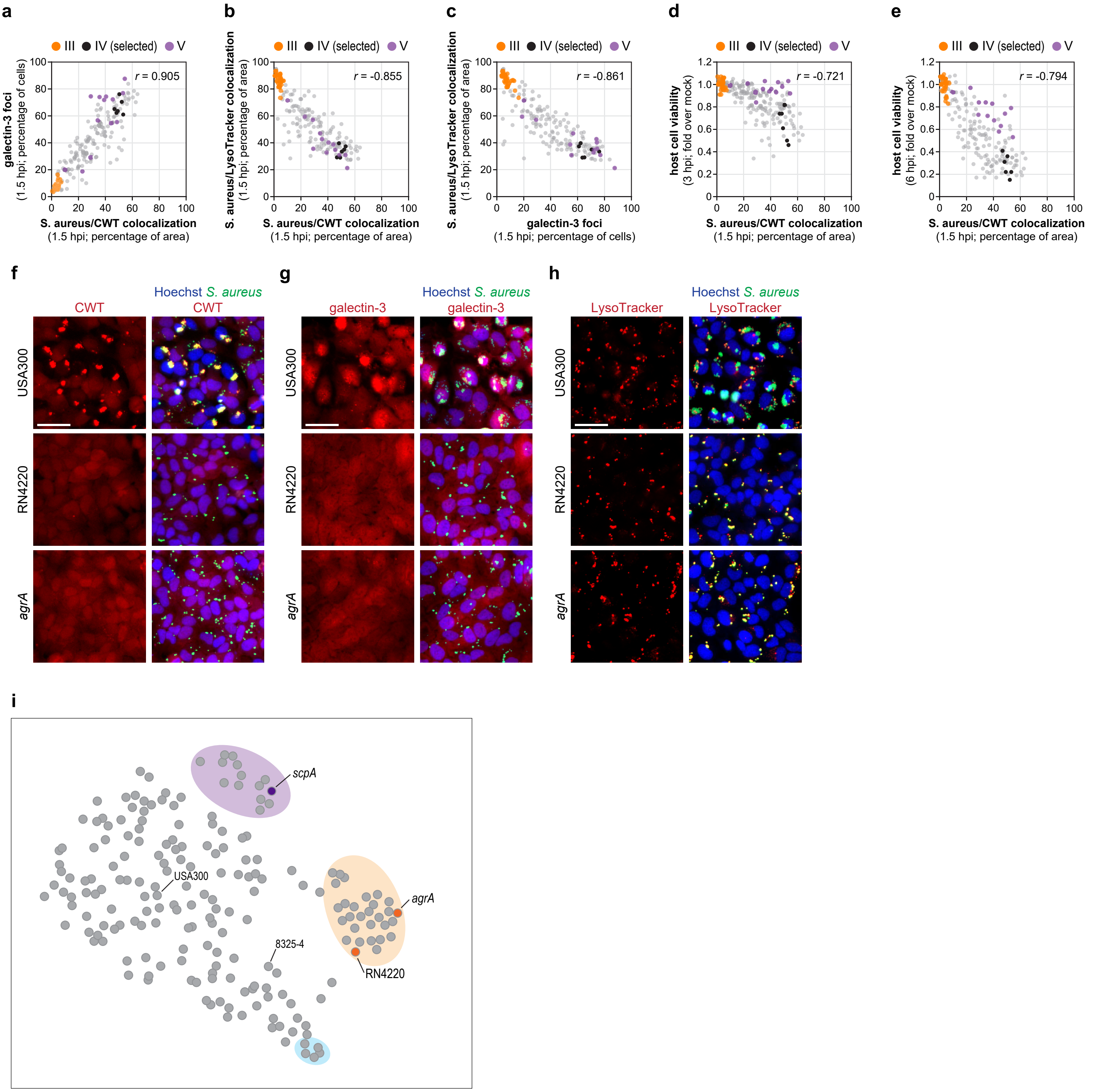


**Supplementary Fig. 8. Vacuolar escape of *S. aureus* isolates correlates with induction of host cell death.**
**a-e.** Pairwise comparison of *S. aureus*/CWT colocalization with percentage of infected cells positive for galectin-3 foci (**a**), or *S. aureus*/LysoTracker colocalization (**b**), percentage infected cells positive for galectin-3 foci with *S. aureus*/LysoTracker colocalization (**c**), or *S. aureus*/CWT colocalization with host cell viability at 3 (**d**) or 6 hpi (**e**). Results shown are the mean of 3 biologically independent experiments. Group III and V bacterial isolates are highlighted in orange and purple, respectively; 6 selected isolates showing high replication and high host cell death (selected from clusters IVa, IVb, IVc) are highlighted in black; all the other isolates are shown in light gray. Spearman's rank correlation coefficients are shown in the upper right corner of each graph.
**f-h.** Representative fluorescence microscopy images of HeLa cells infected with control strains *S. aureus* USA300, RN4220, and *agrA* mutant to evaluate the recruitment of fluorescently labeled CWT (**f**), formation of galectin-3 foci (**g**), and colocalization with LysoTracker (**h**), analyzed at 1.5 hpi. Microscopy images are representative of 3 biologically independent experiments. Scale bar, 50 µm.
**i.** Localization of the *S. aureus* strains RN4220, *agrA*, and *scpA* mutants in the t-SNE visualization of the 191 *S. aureus* isolates based on their individual phenotypic profiles, as described in the legend to **Fig. 4a**.

**Rodrigues Lopes et al. - Supplementary Figure 9**

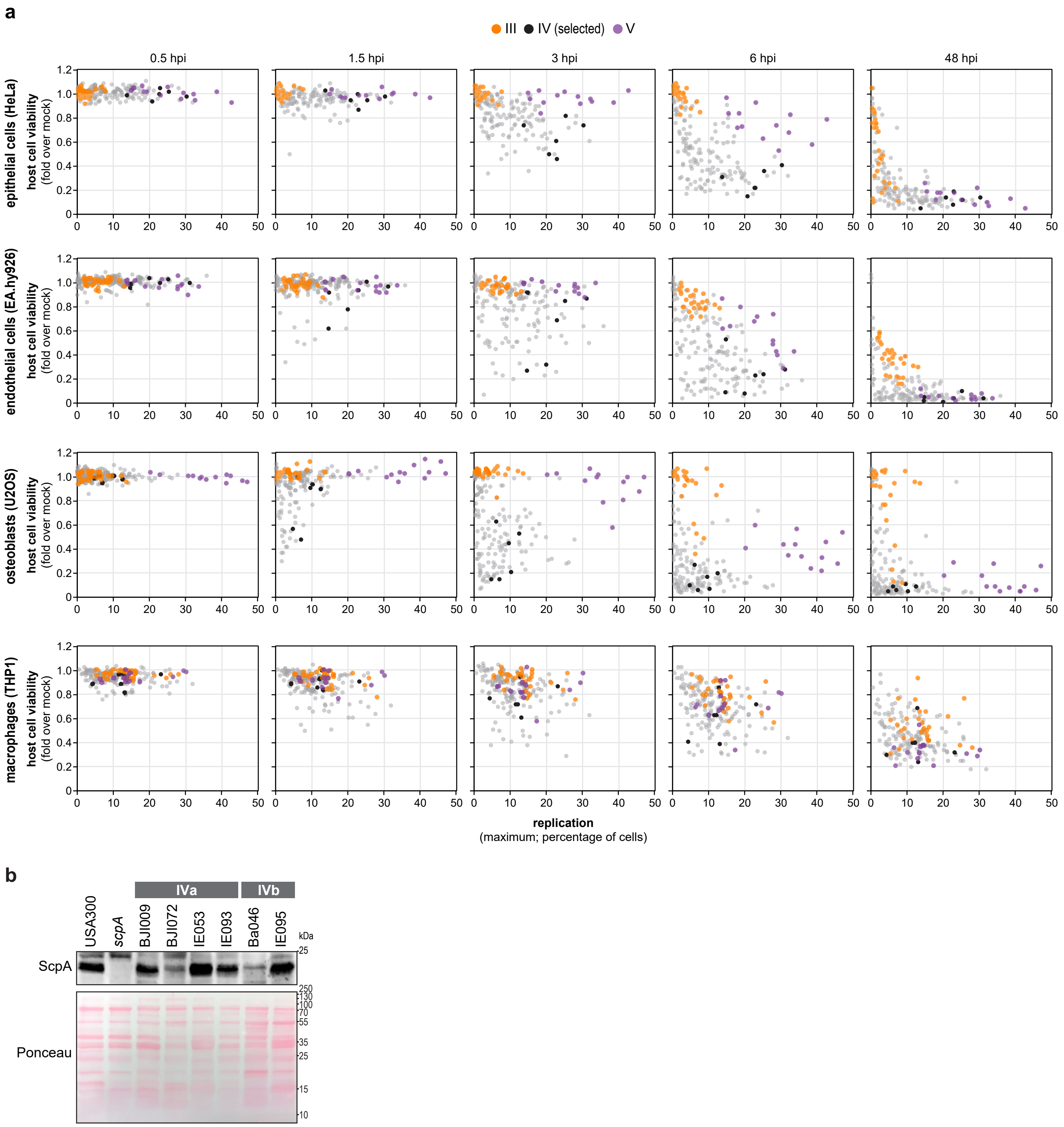


**Supplementary Fig. 9. Intracellular replication of *S. aureus* isolates inversely correlates with host cell viability.**
**a.** Comparison between the percentage of cells with high *S. aureus* intracellular replication (maximum value) with the host cell viability upon infection of the 4 host cell types with the 191 *S. aureus* isolates, analyzed at 0.5, 1.5, 3, 6, and 48 hpi. Results shown are the mean of 3 biologically independent experiments. Group III and V bacterial isolates are highlighted in orange and purple, respectively; 6 isolates selected from clusters IVa, IVb, IVc are highlighted in black; all the other isolates are shown in light gray.
**b.** Staphopain A protein levels, determined by western blot, in the supernatant of bacterial liquid cultures of 6 *S. aureus* isolates belonging to groups IVa (BJI009, BJI072, IE053, IE093) and IVb (Ba046, IE095). *S. aureus* USA300 and *scpA* mutant were used as controls. Western blots are representative of 3 independent experiments. Ponceau staining of the membrane is shown.
Source data are provided as a Source Data file.

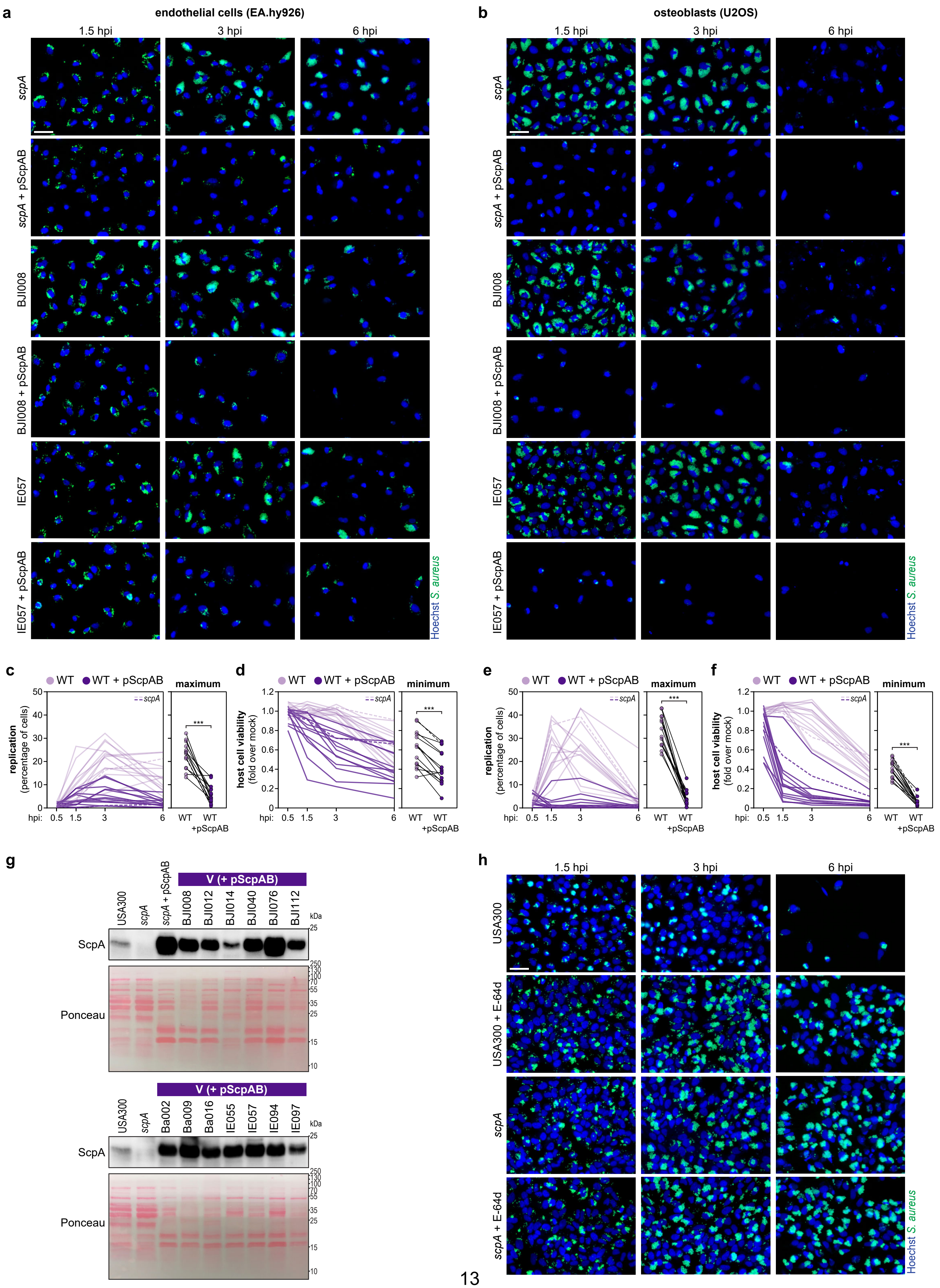
a
endothelial cells (EA.hy926)
1.5 hpi
3 hpi
6 hpi
scpA
scpA + pScpAB
BJI008
BJI008 + pScpAB
IE057
IE057 + pScpAB
Hoechst S. aureus
b
osteoblasts (U2OS)
c
WT
WT + pScpAB
maximum
replication (percentage of cells)
scpA
hpi:
***
d
minimum
host cell viability (fold over mock)
e
f
g
V (+ pScpAB)
USA300
BJI008
BJI012
BJI014
BJI040
BJI076
BJI112
kDa
ScpA
Ponceau
Ba002
Ba009
Ba016
IE055
IE094
IE097
h
USA300 + E-64d
scpA + E-64d

**Supplementary Fig. 10. Ectopic expression of staphopain A in group V *S. aureus* clinical isolates results in impaired intracellular replication and increased host cell death.**

**a and b.** Representative fluorescence microscopy images of endothelial cells (EA.hy926; **a**) or osteoblasts (U2OS; **b**) infected with selected *S. aureus* isolates belonging to cluster V (BJI008 and IE057; WT), and the corresponding isolates ectopically expressing both staphopain A and staphostatin A from a plasmid (WT + pScpAB). *S. aureus scpA* mutant strain and complemented mutant (*scpA* + pScpAB) are shown for comparison. Microscopy images are representative of 3 biologically independent experiments. Scale bar, 50 μm.

**c-f.** Time course quantification of the percentage of cells with high *S. aureus* intracellular replication (**c and e**) and host cell viability (**d and f**) upon infection of endothelial cells (EA.hy926; **c and d**) or osteoblasts (U2OS; **e and f**) with the 13 *S. aureus* isolates belonging to cluster V (WT, light purple) or with the corresponding isolates expressing both staphopain A and staphostatin A from a plasmid (WT + pScpAB, dark purple). *S. aureus scpA* mutant and *scpA* + pScpAB are shown for comparison (dotted lines). Results shown are the mean of 3 biologically independent experiments; rightmost plots indicate the maximum value of replication (**c and e**) or minimum value of host cell viability (**d and f**) for WT or modified bacteria; ***P < 0.001 (statistical analysis is detailed in **Supplementary Data 3**)

**g.** Staphopain A protein levels, determined by western blot, in the supernatant of bacterial liquid cultures of the 13 *S. aureus* isolates belonging to group V ectopically expressing both staphopain A and staphostatin A from a plasmid (+ pScpAB). *S. aureus* USA300, *scpA* mutant, and *scpA* + pScpAB were used as controls. Western blots are representative of 3 independent experiments. Ponceau staining of the membranes is shown.

**h.** Representative fluorescence microscopy images of HeLa cells infected with control strains *S. aureus* USA300 and *scpA* mutant without or with treatment with the inhibitor of cysteine proteases E-64d. Microscopy images are representative of 3 biologically independent experiments. Scale bar, 50 μm.

Source data are provided as a Source Data file.
