# Supplementary material for: Microscopy-based phenotypic profiling of infection by Staphylococcus aureus clinical isolates reveals intracellular lifestyle as a prevalent feature
Source: Nat Commun. 2022 Nov 22;13:7174. doi: 10.1038/s41467-022-34790-9 (PMC9684519; doi:10.1038/s41467-022-34790-9)
Supplement: Supplementary file 3 — Description of Additional Supplementary Files [file 41467_2022_34790_MOESM3_ESM.pdf]

Description of Additional Supplementary Files:

Supplementary Data 1: Complete results from the screening to characterize the intracellular lifestyle of 191 S. aureus isolates. Results for the time course (0.5, 1.5, 3, 6, and 48 hpi) analysis of infection, intracellular replication, and host cell viability for the 191 S. aureus isolates in 4 host cell types, specifically epithelial cells (HeLa), endothelial cells (EA.hy926), osteoblasts (U2OS), and macrophages (differentiated THP1). Information about the clonal complex (CC), sequence type (ST), agr type, phenotypic group of each clinical isolate, and OD600 of the bacterial growths used for infection are shown. Results are shown for the 3 biologically independent experiments, corresponding means, maximum mean values of S. aureus intracellular replication, and minimum mean values of host cell viability. Viability results are shown normalized to mock-treated cells; number of mock treated cells for each independent run of the screening, cell type and time postinfection is also shown. Spearman's rank correlation coefficients between the independent runs of the screening for all phenotypes, cell types and time-points analyzed are also presented.

Supplementary Data 2: Results for the cytosolic/vacuolar localization of the 191 S. aureus isolates. Results for percentage of cells with high S. aureus intracellular replication (maximum value), percentage of S. aureus/CWT colocalization (at 1.5 hpi), percentage of infected cells positive for galectin-3 foci (at 1.5 hpi), and percentage of S. aureus/LysoTracker colocalization (at 1.5 hpi), for epithelial cells (HeLa) infected with the 191 S. aureus isolates. Results are shown for the 3 biologically independent experiments and corresponding means.

Supplementary Data 3: Statistical analyses.

Supplementary Video 1: Timelapse microscopy (24 h) of HeLa cells infected with an S. aureus clinical isolate belonging to cluster III (BJI035).

Supplementary Video 2: Timelapse microscopy (24 h) of HeLa cells infected with an S. aureus clinical isolate belonging to cluster IVa (BJI009).

Supplementary Video 3: Timelapse microscopy (24 h) of HeLa cells infected with an S. aureus clinical isolate belonging to cluster V (BJI008).

Supplementary Video 4: Timelapse microscopy (24 h) of HeLa cells infected with S. aureus USA300.
