# Supplementary material for: Microscopy-based phenotypic profiling of infection by Staphylococcus aureus clinical isolates reveals intracellular lifestyle as a prevalent feature
Source: Nat Commun. 2022 Nov 22;13:7174. doi: 10.1038/s41467-022-34790-9 (PMC9684519; doi:10.1038/s41467-022-34790-9)
Supplement: Supplementary file 11 — Reporting Summary [file 41467_2022_34790_MOESM11_ESM.pdf]

nature portfolio

Corresponding author(s): Ana Eulalio
Miguel Mano

Last updated by author(s): Nov 3, 2022

Reporting Summary

Nature Portfolio wishes to improve the reproducibility of the work that we publish. This form provides structure for consistency and transparency in reporting. For further information on Nature Portfolio policies, see our Editorial Policies and the Editorial Policy Checklist.

Statistics

For all statistical analyses, confirm that the following items are present in the figure legend, table legend, main text, or Methods section.

n/a	Confirmed	
	x	The exact sample size (*n*) for each experimental group/condition, given as a discrete number and unit of measurement
	x	A statement on whether measurements were taken from distinct samples or whether the same sample was measured repeatedly
	x	The statistical test(s) used AND whether they are one- or two-sided<br>*Only common tests should be described solely by name; describe more complex techniques in the Methods section.*
x		A description of all covariates tested
	x	A description of any assumptions or corrections, such as tests of normality and adjustment for multiple comparisons
	x	A full description of the statistical parameters including central tendency (e.g. means) or other basic estimates (e.g. regression coefficient) AND variation (e.g. standard deviation) or associated estimates of uncertainty (e.g. confidence intervals)
	x	For null hypothesis testing, the test statistic (e.g. *F*, *t*, *r*) with confidence intervals, effect sizes, degrees of freedom and *P* value noted<br>*Give P values as exact values whenever suitable.*
x		For Bayesian analysis, information on the choice of priors and Markov chain Monte Carlo settings
x		For hierarchical and complex designs, identification of the appropriate level for tests and full reporting of outcomes
x		Estimates of effect sizes (e.g. Cohen's *d*, Pearson's *r*), indicating how they were calculated

Our web collection on statistics for biologists contains articles on many of the points above.

Software and code

Policy information about availability of computer code

Data collection	Harmony (PerkinElmer, v.4.1), EnSpire Manager (PerkinElmer, v.413.3005.1482)
Data analysis	Columbus image analysis software (PerkinElmer, v.2.7.1), Microsoft Excel (Microsoft Office, 2013), Prism (GraphPad, v.7.00), Adobe Photoshop (Adobe, CS6, CC), Adobe Illustrator (Adobe, CS6, CC), TIBCO Spotfire Analyst (v.10.10.3; TIBCO), Interactive t-SNE web tool (https://jefworks.github.io/tsne-online/), Cutadapt (v3.4), Trimmomatic (v0.39), SPAdes (v3.13.0), QUAST (v5.0.2), Prokka (v1.14.5), Roary (v3.13.0), FastTree (v2.1.10), SAMtools (v1.15), Blastn (v2.12.0+), MAFFT(v7), SankeyMATIC web tool (https://sankeymatic.com/)

For manuscripts utilizing custom algorithms or software that are central to the research but not yet described in published literature, software must be made available to editors and reviewers. We strongly encourage code deposition in a community repository (e.g. GitHub). See the Nature Portfolio guidelines for submitting code & software for further information.

Data

Policy information about availability of data

All manuscripts must include a data availability statement. This statement should provide the following information, where applicable:

- Accession codes, unique identifiers, or web links for publicly available datasets
- A description of any restrictions on data availability
- For clinical datasets or third party data, please ensure that the statement adheres to our policy

The data corresponding to the S. aureus phenotypic screenings are provided in supplementary information (Supplementary Data 1 and 2); raw microscopy datasets and custom image analysis workflows implemented in Columbus image analysis software are available from the corresponding authors upon reasonable request; the S. aureus clinical isolates genome sequencing data have been deposited in the European Nucleotide Archive (ENA), accession number PRJEB48298, available at https://www.ebi.ac.uk/ena/browser/view/PRJEB48298. The genome sequences or agr locus sequence of S. aureus strains used in this study are available in the

National Center for Biotechnology Information (NCBI) GenBank under accession numbers NC_007795.1 (S. aureus 8325; https://www.ncbi.nlm.nih.gov/nuccore/NC_007795), CP000046.1 (S. aureus COL; https://www.ncbi.nlm.nih.gov/nuccore/CP000046), BA000017.4 (S. aureus Mu50; https://www.ncbi.nlm.nih.gov/nuccore/BA000017.4), BX571856.1 (S. aureus MRSA252; https://www.ncbi.nlm.nih.gov/nuccore/BX571856.1), and AF288215.1 (S. aureus RN5881; https://www.ncbi.nlm.nih.gov/nuccore/AF288215.1). Source data are provided with this paper.

Field-specific reporting

Please select the one below that is the best fit for your research. If you are not sure, read the appropriate sections before making your selection.

[x] Life sciences [ ] Behavioural & social sciences [ ] Ecological, evolutionary & environmental sciences

For a reference copy of the document with all sections, see nature.com/documents/nr-reporting-summary-flat.pdf

Life sciences study design

All studies must disclose on these points even when the disclosure is negative.

Sample size	No statistical methods were used to predetermine sample size. The sample size was chosen to include at least 3 biologically independent experiments. Sample size was based on standard sample sizes from our past experiments and similarly to what is described for similar experiments in published articles (cf. Maudet et al. Nat. Commun. 2014 5:4718; Aguilar & Cruz et al. Nat Microbiol. 2020 5:192)
Data exclusions	No data were excluded from the analysis.
Replication	Experiments were independently performed at least 3 times, and all attempts of replication were successful.
Randomization	Animals were randomly assigned to experimental groups. For in vitro experiments, cultured cells were uniformly plated, with random allocation of treatment.
Blinding	Blinding was not performed in this study. Image acquisition and analysis are automated, and independent of the researcher performing the experiment.

Reporting for specific materials, systems and methods

We require information from authors about some types of materials, experimental systems and methods used in many studies. Here, indicate whether each material, system or method listed is relevant to your study. If you are not sure if a list item applies to your research, read the appropriate section before selecting a response.

Materials & experimental systems

n/a	Involved in the study
[ ]	[x] Antibodies
[ ]	[x] Eukaryotic cell lines
[x]	[ ] Palaeontology and archaeology
[ ]	[x] Animals and other organisms
[x]	[ ] Human research participants
[x]	[ ] Clinical data
[x]	[ ] Dual use research of concern

Methods

n/a	Involved in the study
[x]	[ ] ChIP-seq
[x]	[ ] Flow cytometry
[x]	[ ] MRI-based neuroimaging

Antibodies

Antibodies used	rabbit polyclonal anti-staphopain A antibody (1:1,000; antibodies online, ABIN967004; RRID:AB_2894409), anti-rabbit IgG coupled to horseradish peroxidase (1:10,000, GE Healthcare, NA934)
Validation	anti-staphopain A antibody was validated in our study by western-blot in samples of S. aureus insertional transposon mutants of scpA (Figure 6C and S7G)

Eukaryotic cell lines

Policy information about cell lines

Cell line source(s)	HeLa-229 (ATCC, CCL-2.1), U2OS (ATCC, HTB-96), EA.hy926 (ATCC, CRL-2922), THP1 (ATCC, TIB-202), and HEK293T (ATCC, CRL-3216) were acquired from ATCC/LGC Standards. HeLa-229 cells stably expressing mCherry-Galectin3 and HeLa-229 cells stably expressing mRFP-CWT escape marker were generated in the Eulalio and Mano laboratories, as described in the Methods section. Human umbilical vein endothelial cells (HUVEC; Lonza, C2519A) were acquired from Lonza.

Authentication	None of the cell lines was authenticated.
Mycoplasma contamination	All cells were tested and negative for mycoplasma contamination.
Commonly misidentified lines (See ICLAC register)	No commonly misidentified cell lines were used in this study.

Animals and other organisms

Policy information about studies involving animals; ARRIVE guidelines recommended for reporting animal research

Laboratory animals	Final instar larval stage Galleria mellonella (250-300 mg) were purchased from UK Waxworms.
Wild animals	The study did not involve wild animals.
Field-collected samples	The study did not involve field collected samples.
Ethics oversight	The work involving invertebrates does not need ethical permission.

Note that full information on the approval of the study protocol must also be provided in the manuscript.
